# No association between temozolomide maintenance and survival after first‐line treatment in primary CNS lymphoma

**DOI:** 10.1002/hem3.70445

**Published:** 2026-08-17

**Authors:** Nashif Chowdhury, Jenni Niemi, Haris Babačić, Hanna‐Maija Rantakylö, Ingrid Glimelius, Mats Jerkeman, Mats Hellström

**Affiliations:** ^1^ Department of Immunology, Genetics and Pathology Uppsala University Uppsala Sweden; ^2^ Department of Oncology and Pathology Karolinska Institute and Science for Life Laboratory Stockholm Sweden; ^3^ Department of Clinical Sciences Lund, Division of Oncology Skåne University Hospital, Lund University Lund Sweden

Primary central nervous system lymphoma (PCNSL) is a rare and aggressive form of diffuse large B‐cell lymphoma (DLBCL) characterized by infiltration of malignant B cells into the central nervous system (CNS).^1^ Fit patients are treated with high‐dose methotrexate (HD‐MTX)–based polychemotherapy followed by thiotepa‐containing high‐dose chemotherapy and autologous stem cell transplantation (ASCT).^2^ For patients unsuitable for ASCT, induction treatment is consolidated with high‐dose cytarabine and low‐dose whole‐brain radiotherapy (WBRT).^1^, ^2^ Patients unsuitable for HD‐MTX can receive first‐line treatment with a single alkylator, such as temozolomide (TMZ) and WBRT. Despite therapeutic advances and high response rates, over half of the patients with PCNSL relapse within 2 years after diagnosis.^3^ TMZ maintenance treatment has been suggested to reduce relapse rates and improve overall survival (OS) for patients with post‐induction complete remission (CR) or partial remission (PR). Therefore, its administration is supported by international guidelines, including the European Hematology Association and European Society for Medical Oncology (EHA‐ESMO) joint recommendations^2^ and the National Comprehensive Cancer Network (NCCN) Guidelines for B‐Cell Lymphomas (Version 3.2026). However, implementation of TMZ maintenance varies across clinical settings, allowing real‐world evaluation of the association between TMZ maintenance and OS.

To address the clinical evidence gap regarding TMZ maintenance benefits, we aimed to evaluate its OS gain in a retrospective cohort of 110 patients with PCNSL diagnosed between 2009 and 2022 at two Swedish university hospitals: Uppsala University Hospital (*n* = 60) and Skåne University Hospital in Lund (*n* = 50), respectively. The Uppsala subgroup was included from the U‐CAN population‐based prospective biobank.^4^, ^5^ We collected information on patient characteristics, treatments, and outcomes. We performed univariable survival analyses with Kaplan–Meier curves and time‐dependent Cox proportional hazards models, tested with two‐sided log‐rank and Wald tests, respectively, at a statistical significance threshold of *α* = 0.05. OS was the primary outcome, defined as the time from diagnosis to death or last vital status assessment. To adjust for the effects of different treatments and account for potential immortal time bias, we performed multivariable analyses with time‐dependent Cox proportional hazards models by introducing the treatment effects on OS at the time of treatment start, whereas CR was introduced at the end of induction. Study design and methodology are available in Supporting Information S2: Supplementary Methods.

In the entire cohort, 84 patients (76%) received HD‐MTX, mainly the MPV (HD‐MTX, procarbazine, and vincristine) regimen, given to 62 of 110 patients (56%). In addition to HD‐MTX, patients received a variety of other first‐line treatments: 14 patients (13%) underwent ASCT, 38 patients (35%) WBRT, and 21 patients (19%) TMZ during induction (Supporting Information S1: Table 1). TMZ maintenance was offered on an individual basis to patients with CR or PR after induction therapy, particularly those who were not candidates for intensive consolidation, for example, ASCT.^2^ By the end of follow‐up, 78 patients (71%) had deceased, with a median OS for the full cohort of 29.1 months (Supporting Information S1: Figure 1A). As expected, age and higher Eastern Cooperative Oncology Group (ECOG) performance status score were strongly associated with worse OS (Supporting Information S1: Figure 1B,C), whereas sex had no impact on OS (Supporting Information S1: Figure 1D). Furthermore, OS differed according to treatments in univariable Kaplan–Meier analyses (Supporting Information S1: Figure 2A–E). HD‐MTX had the greatest effect, with patients receiving it showing longer OS than those who did not (median OS: 55.8 vs. 2.2 months; ranges: 1.1–201.0 and 0.3–98.3 months, respectively; Supporting Information S1: Figure 2A). Patients achieving CR after first‐line induction had longer OS than patients with PR, stable disease (SD), or progressive disease (PD) (Supporting Information S1: Figure 2F). Taken together, OS in the present cohort was primarily driven by established prognostic and treatment‐related factors.

One third of patients (*n* = 34, 31%) received TMZ maintenance, administered according to institutional practice as oral TMZ for 5 days, every 28 days. The patients completed a median of 10 cycles (range: 1–13), of whom 20 patients discontinued treatment due to progression or toxicity. Patients receiving TMZ maintenance had similar characteristics to patients without TMZ maintenance, with a non‐statistically significant trend to more rituximab treatment and WBRT in the TMZ maintenance group (Table 1). In univariable analyses, patients who received TMZ maintenance at any point during follow‐up had longer OS (P = 0.043), with a 5‐year OS of 50% (95% confidence interval [CI], 35.7–70) compared with 38.1% (95% CI, 28.6–50.7) in patients without TMZ maintenance (Figure 1A). Maintenance treatment is, by definition, initiated after a variable period after diagnosis, introducing a risk of immortal time bias where patients on TMZ maintenance artificially gain follow‐up survival time if maintenance exposure is considered from the date of diagnosis. In line with this, we noted that the timing of TMZ maintenance initiation varied substantially between patients (Figure 1B). To adjust for the onset of TMZ maintenance treatment effect and exclude the survival time prior to treatment start, we treated TMZ maintenance as a time‐dependent variable in univariable Cox models. This time‐dependent analysis showed no association between TMZ maintenance and OS (hazard rate ratio [HR], 0.944, 95% CI, 0.542–1.643, P = 0.839). The estimates were similar when stratifying for treatment centers, with HR of 1.042 (95% CI, 0.437–2.486, P = 0.926) and 0.825 (95% CI, 0.399–1.705, P = 0.603) for Lund and Uppsala, respectively. Since several other confounders could have affected the association between TMZ maintenance and OS, we tested thirteen different variables for their association with OS in a time‐dependent multivariable Cox model, including age <50 years, sex, first‐line treatments, completion of HD‐MTX treatment (≥3 treatment cycles), MPV regimen, MATRix regimen, rituximab treatment, treatment response (CR status), deep structures involvement, and treatment center. ECOG performance status was not considered in the main analysis due to the large proportion of missing values (*n* = 31, 28%). Most variables, excluding age, sex, MPV and MATRix regimens, rituximab, and involvement of deep structures, were associated with OS at P < 0.1, and we adjusted the association between TMZ maintenance and OS for these confounders, which recapitulated no association between TMZ maintenance and OS (Figure 1C). This observation was also supported by Kaplan–Meier analyses when analyzing only patients who completed HD‐MTX and stratified for TMZ maintenance with or without WBRT or taking treatment response into account (Supporting Information S1: Figure 3A–C). Since the Uppsala and Lund patients differed in ECOG performance status (Supporting Information S1: Table 1), we performed a sensitivity analysis on patients with reported ECOG performance status score (*n* = 79) by introducing this variable in the multivariable model as well, which reproduced the lack of association between TMZ maintenance and OS (Figure 1D). In addition, we observed no difference in OS between the centers in the sensitivity analysis, likely due to the confounding effect of ECOG performance status on OS in the Lund subgroup. Thus, after adjusting for treatment initiation and the time‐dependent confounding effects of other treatments, TMZ maintenance showed no evidence for association with OS.

**Table 1:** Patient characteristics according to temozolomide maintenance.

		All	Temozolomide maintenance	No temozolomide maintenance	P‐value
Number of patients		110	34	76	
Sex: male (%); female (%)		58 (53); 52 (47)	16 (47); 18 (53)	42 (55); 34 (45)	0.7
Median age in years (range, standard deviation)		67 (29–82, 10.6)	67 (45–81, 7.8)	65 (29–82, 11.8)	0.7
Age groups per years, *N* (%)	29–49	10 (9)	1 (3)	9 (12)	
	50–59	13 (12)	5 (15)	8 (11)	
	60–69	45 (41)	15 (44)	30 (39)	
	≥70	42 (38)	13 (38)	29 (38)	0.4
Site of disease (%^a^)	Deep structures	77 (73)	23 (72)	54 (71)	1.0
	Unknown	4	3	1	
ECOG performance status (WHO) (%^a^)	0	24 (30)	8 (27)	16 (33)	
	1	36 (46)	15 (50)	21 (43)	
	2	7 (9)	3 (10)	4 (8)	
	3	8 (10)	4 (13)	4 (8)	
	4	4 (5)	0 (0)	4 (8)	0.5
	Unknown	31	4	27	
MSKCC score (%^a^)	1	5 (6)	0 (0)	5 (10)	
	2	56 (71)	24 (80)	32 (65)	
	3	18 (23)	6 (20)	12 (24)	0.2
	Unknown	31	4	27	
HD‐MTX regimen *N* (%^a^)^b^	MATRix	11 (10)	3 (11)	8 (14)	
	MPV	62 (56)	22 (78)	40 (71)	
	MATRix + MPV	5 (5)	2 (7)	3 (5)	
	Other	6 (5)	1 (4)	5 (9)	0.6
Rituximab^b^		60 (55)	23 (67)	37 (49)	0.1
WBRT^b^		38 (35)	16 (47)	22 (29)	0.08
ASCT		14 (13)	4 (12)	10 (13)	1.0
1st line induction response (%^a^)	CR	51 (53)	17 (57)	34 (52)	
	PR	23 (24)	10 (33)	13 (20)	
	SD	6 (6)	3 (10)	3 (5)	
	PD	7 (7)	0 (0)	7 (11)	0.13^c^
	(Not applicable^d^)	9 (9)	0 (0)	9 (12)	
	Unknown	14	4	10	
Median number of temozolomide maintenance cycles (range)			10 (1–13)	0 (0)	

*Note*: All patients (*n* = 110, left column) were stratified according to receipt of temozolomide maintenance treatment (middle column) or no temozolomide maintenance treatment (right column). Percentages are presented in parentheses.

Abbreviations: ASCT, autologous stem cell transplantation; CR, complete response; ECOG, Eastern Cooperative Oncology Group; HD‐MTX, high‐dose methotrexate; MATRix, methotrexate, cytarabine, thiotepa, and rituximab; MPV, methotrexate, procarbazine, and vincristine; MSKCC, Memorial Sloan Kettering Cancer Center score; *n*, number; PD, progressive disease; PR, partial response; SD, stable disease; WBRT, whole‐brain radiotherapy; WHO, World Health Organization.

^a^ Percentage from patients with known status.

^b^ As a part of the primary treatment.

^c^ P‐value refers to the comparison of radiological responses (CR, PR, SD, and PD).

^d^ Received no active treatment and hence no radiological evaluation.

**Figure 1:**
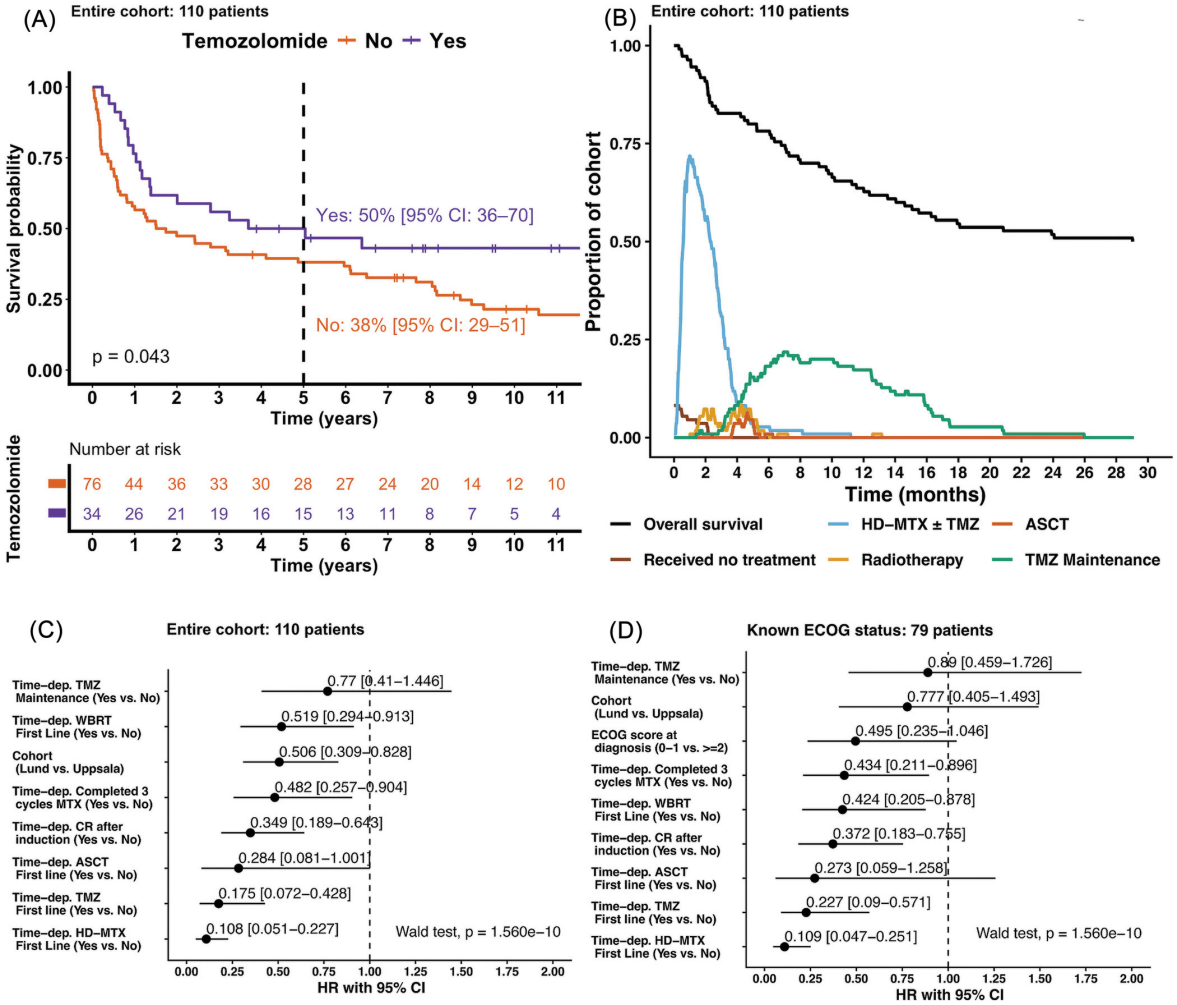
**Primary central nervous system lymphoma (PCNSL) treatment heterogeneity and survival analysis of temozolomide (TMZ) maintenance. (A)** Overall survival stratified according to TMZ maintenance in the entire study population. **(B)** Prevalence of treatment administration over time (*x*‐axis) in the study population (*n* = 110), censored at the median survival time of 29.1 months. The last administered treatment (TMZ maintenance) was discontinued 26 months after diagnosis. **(C)** Cox model showing the association between TMZ maintenance and overall survival, accounting for time‐dependent effects and adjusted for confounders with an effect on overall survival at P < 0.1. **(D)** Sensitivity analysis of the time‐dependent Cox model showing the association between TMZ maintenance and overall survival, further adjusted for ECOG performance status in addition to other confounders. Abbreviations: ASCT, autologous stem cell transplantation; CR, complete response; ECOG, Eastern Cooperative Oncology Group; HD‐MTX, high‐dose methotrexate; HR, hazard rate ratio; MTX, methotrexate; Time‐dep, time‐dependent; WBRT, whole‐brain radiotherapy.

This study assessed the effect of TMZ maintenance on OS while accounting for first‐line treatments and variability of treatment timing. TMZ maintenance was partly adopted after the excellent outcome of two single‐arm studies. The first study employed TMZ maintenance after HD‐MTX in combination with first‐line TMZ induction treatment followed by WBRT consolidation.^6^ The second study evaluated elderly patients (>65 years) whose first‐line treatment included HD‐MTX, cytarabine, and TMZ, followed by TMZ maintenance.^7^ Notably, in both studies, TMZ maintenance was embedded within complex, multimodal treatment strategies including TMZ within first‐line induction treatment, making it difficult to isolate the specific effect of TMZ maintenance. A retrospective US cohort of 539 patients supported maintenance treatment in PCNSL, but many patients received other agents than TMZ, such as lenalidomide or methotrexate, and did not correct for immortal time bias.^8^ However, a recent randomized Phase III trial (ID: JCOG1114C) of 122 Japanese patients treated with HD‐MTX induction and WBRT failed to demonstrate a survival benefit associated with TMZ maintenance.^9^ Approximately 87% and 71% of the patients were alive 2 years after treatment randomization in the non‐TMZ and TMZ arms, respectively, without significant survival differences in subgroup analyses of radiological responses. In this context, it is also important to consider that any maintenance treatment comes with a burden of toxicity, including fatigue, increased risk of infections, and hematological adverse events. In the JCOG1114C trial, Grade 3–4 lymphopenia almost doubled to 44% in the TMZ maintenance arm, potentially affecting infection risk, disease control, and survival. Taken together, the available data suggest that TMZ is well tolerated and effective in PCNSL patients when combined with WBRT or HD‐MTX–based therapies, though evidence supporting its use as maintenance treatment in patients remains limited.

Strengths of this study include the population‐based two‐center design, comprehensive clinical data collection, long follow‐up, and analyses accounting for confounders and time‐dependent effects. Limitations include the retrospective design, treatment heterogeneity, non‐standardized criteria for maintenance treatment, limited sample size, and incomplete data capture.

In summary, this real‐world two‐center study found no consistent association between TMZ maintenance and OS after adjustment for confounders and time‐dependent effects. Together with emerging evidence from a randomized clinical trial, these findings suggest that the role of TMZ maintenance in routine clinical practice warrants reconsideration.

## AUTHOR CONTRIBUTIONS

**Nashif Chowdhury**: Conceptualization; investigation; writing—original draft; methodology; visualization; writing—review and editing; software; formal analysis; project administration; data curation; resources; validation. **Jenni Niemi**: Conceptualization; investigation; writing—original draft; methodology; visualization; writing—review and editing; software; formal analysis; project administration; data curation; resources; validation. **Haris Babačić**: Writing—original draft; methodology; visualization; writing—review and editing; software; formal analysis; data curation; investigation; validation; project administration; resources. **Hanna‐Maija Rantakylö**: Investigation; formal analysis; data curation. **Ingrid Glimelius**: Conceptualization; writing—original draft; writing—review and editing; methodology; validation; formal analysis; supervision; project administration; visualization; investigation. **Mats Jerkeman**: Conceptualization; investigation; writing—original draft; writing—review and editing; methodology; validation; formal analysis; supervision; funding acquisition; project administration; visualization. **Mats Hellström**: Conceptualization; investigation; funding acquisition; writing—original draft; methodology; validation; visualization; writing—review and editing; formal analysis; project administration; data curation; supervision; resources; software.

## CONFLICT OF INTEREST STATEMENT

N.C., J.N., H.B., and H.‐M. R. declare no conflicts of interest. M.J. has received honoraria from AbbVie, AstraZeneca, BeOne, BMS, Galapagos, Janssen, Lilly, Roche, Kite Gilead, and Takeda, and research funding from AbbVie, AstraZeneca, and Roche. M.H. has received lecture honoraria from Roche, Kite Gilead, BMS, and Incyte; has served on advisory boards for Incyte, Kite Gilead, and Kyowa Kirin; and has received research funding from Novartis. I.G. has received lecture honoraria to the department from Kite Gilead, AbbVie, Takeda, and Roche, and has participated in real‐world data collection with Takeda.

## ETHICS STATEMENT

This study was conducted in accordance with the Declaration of Helsinki and was approved by the Swedish Ethical Review Authority (Sw: Etiksprövningsmyndigheten, EPM), for Uppsala University Hospital (approval number 2022‐02857‐01) and for Skåne University Hospital (approval number 2020‐05582).

## FUNDING

This work was supported by the Swedish Cancer Society (M.H: 24 3827 Pj and I.G: 25 4431 Pj 01H) and ALF funds at Uppsala University Hospital.

## Supporting information

Supporting Information.

Supporting Information.

## Data Availability

The data that support the findings of this study are available on request from the corresponding author. The data are not publicly available due to privacy or ethical restrictions.
