# Supplementary material for: No association between temozolomide maintenance and survival after first‐line treatment in primary CNS lymphoma
Source: Hemasphere. 2026 Aug 17;10(8):e70445. doi: 10.1002/hem3.70445 (PMC13479465; doi:10.1002/hem3.70445)
Supplement: Supplementary file 1 [file HEM3-10-e70445-s001.docx]

**Supplementary methods**

**Study design and data**

This is a two-center retrospective cohort study using routinely collected clinical data from PCNSL (primary CNS lymphoma) patients diagnosed between 2009 and 2022 in Sweden. Eligibility criteria for inclusion were histologically confirmed diffuse large B-cell lymphoma and exclusive CNS localization. Patients were treated in accordance with Swedish and European guidelines (**Suppl.** **Table 1**). The primary endpoint was overall survival (OS), defined as time from diagnosis to death or last vital status assessment. The study reflects routine clinical practice without selection for clinical trial participation.

**Patient cohorts and data**

Pseudonymized data from two cohorts were collected separately and analyzed both independently and together. Medical record data from the first cohort of 60 consecutive patients diagnosed with PCNSL between 2010–2022 were obtained from the Uppsala–Umeå Comprehensive Cancer Consortium (U-CAN) biobank. U-CAN is a population-based biobank that prospectively and consecutively collects a comprehensive set of clinical and biological materials (1), representing cases from the central Sweden healthcare region. Previous analyses of all the B-cell lymphomas in U-CAN showed that cause-specific survival was not significantly different compared to population-representative groups, despite demographic skewing toward fitter patients (2). A second subgroup of patients consisting of 50 PCNSL patients diagnosed during 2009–2018 and treated with HD-MTX at Skåne University Hospital in Lund represented cases from southern Sweden. Information was collected on patient characteristics at the time of diagnosis, underlying comorbidities, treatment modalities, and treatment outcomes.

All patients were followed from the date of diagnosis to date of death or were censored at the date of last vital status assessment, defined as the date on which patient records were reviewed and the patient was confirmed to be alive (data cutoff: April 13, 2026). Patients were retrospectively assigned the Memorial Sloan Kettering Cancer Center (MSKCC) score, a prognostic score based on age and Karnofsky Performance Status (KPS) at diagnosis (3). MSKCC score of 1 included patients aged ≤50 years at diagnosis with KPS ≥70 (equivalent to WHO Performance Status ≤1), MSKCC 2 included patients >50 years with KPS ≥70, and MSKCC 3 comprised patients >50 years old with KPS <70.

**Treatment**

Following Swedish national guidelines, which are aligned with the European Hematology Association and European Society for Medical Oncology (EHA-ESMO) joint recommendations and the National Comprehensive Cancer Network (NCCN) Guidelines for B-Cell Lymphomas (Version 3.2026), curative-intent 3.5 g/m² HD-MTX was offered to patients meeting treatment criteria. HD-MTX was administered within a polychemotherapeutic regimen, either MATRix (HD-MTX, HD-cytarabine, rituximab, thiotepa), MPV (HD-MTX, procarbazine, vincristine), MT (HD-MTX, TMZ), or MTX-AraC (HD-MTX, cytarabine) (4). Following HD-MTX treatment, patients receiving MPV were also offered one to two cycles of high-dose cytarabine (5). Treatment was then consolidated with either WBRT at a minimum dose of 23.4 Gy delivered in 1.8 Gy fractions, or thiotepa-based high-dose chemotherapy with ASCT for younger or fitter patients (4). Primary treatment for patients considered unsuitable for HD-MTX was defined according to Swedish national guidelines as two cycles of rituximab–TMZ followed by WBRT, with or without concomitant TMZ. TMZ maintenance is primarily offered to patients who achieve a complete or partial response (CR/PR) after induction therapy, particularly those who are not candidates for intensive consolidation (e.g., ASCT) (4). TMZ maintenance was generally administered at 150–200 mg/m²/day on days 1–5 every 28 days according to institutional practice. Patients who were not candidates for chemotherapy but were still fit enough to receive active treatment, received WBRT alone. Treatment allocation was based on the treating physician’s assessment and was generally consistent with Swedish national guidelines (**Suppl.** **Table 1**). No protocol-mandated criteria were used to assign patients to specific induction, consolidation, or maintenance strategies.

Radiological assessment of response to induction treatment was generally performed using contrast-enhanced MRI after two cycles and after completion of induction therapy. Response categories were defined according to International Primary CNS Lymphoma Collaborative Group (IPCG) response criteria (6) and categorized as complete response (CR), unconfirmed complete response (Cru), partial response (PR), stable disease (SD), progressive disease (PD), or not assessed. Both CR and CRu were combined in the analysis as CR and were defined as disappearance of all contrast-enhancing disease, PR as at least a 50% reduction, PD as at least a 25% increase and/or appearance of new lesions, and SD as disease not meeting criteria for CR, PR, or PD.

**Statistical analysis**

Descriptive statistics were calculated for all collected and analyzed variables. Categorical data were analyzed using two-sided Fisher’s exact test, and continuous variables were analyzed using two-sided Mann–Whitney U test. Univariable associations between clinical variables and OS were assessed using Kaplan–Meier curves and time-dependent Cox proportional hazards models, tested with two-sided log-rank and Wald tests, respectively, at a statistical significance threshold of α = 0.05. OS was defined as the time from initial lymphoma diagnosis to date of death or last vital status assessment. To adjust for the effects of different treatments on OS, we performed multivariable analyses with time-dependent Cox proportional hazards models. To explore potential confounders, we first tested thirteen clinical variables for their association with OS in a multivariable Cox model: sex (female vs. male), age at diagnosis <50 years old (vs. ≥ 50 years), first-line HD-MTX (yes vs. no), MPV regimen (yes vs. no), MATRix regimen (yes vs. no), first-line rituximab (yes vs. no), first-line WBRT (yes vs. no), first-line ASCT (yes vs. no), first-line TMZ (yes vs. no), completed HD-MTX induction (administered three cycles or more, yes vs. no), CR after induction (yes vs. no or unknown), involvement of deep structures (yes vs. no), and treatment center (Lund vs. Uppsala). To account for immortal time bias, the treatment variables were handled as time-dependent covariates by introducing the treatment’s effect on survival at the time of treatment start and CR was introduced at the time of end of induction. To provide more accurate hazard rate estimates for multiple time observations for individuals with more than one treatment, the observations were clustered per person in the model. After backward stepwise selection of the thirteen variables, removing variables from the model that were above the statistical significance threshold of α = 0.1, the remaining variables were then used to adjust the association between TMZ maintenance and OS. To further assess how patient’s performance status, categorized per the World Health Organization (WHO) Eastern Cooperative Oncology Group (ECOG) scale, affected OS we performed a sensitivity analysis where we further introduced better ECOG performance status (score of 0 or 1 vs. ≥2) in the Cox model. All analyses were conducted with the statistical software R (version 4.5.2), using the packages survminer (0.5.2) and survival (v.3.8-3) for survival analyses and ggplot2 (v.4.0.3) for visualization.

**Ethical considerations**

This study was conducted in accordance with the principles of the Declaration of Helsinki and was approved by the Swedish Ethical Review Board (Sw: Etiksprövningsmyndigheten, EPM), for Uppsala University Hospital (approval number 2022-02857-01) and for Skåne University Hospital (approval number 2020-05582).

**References**

1.	Glimelius B, Melin B, Enblad G, Alafuzoff I, Beskow A, Ahlstrom H, et al. U-CAN: a prospective longitudinal collection of biomaterials and clinical information from adult cancer patients in Sweden. Acta Oncol. 2018;57(2):187–94.

2.	Forsgren E, Ekberg S, Smedby KE, Nylund P, Sjoblom T, Flogegard M, et al. Evaluation of coverage, generalisability and validity of the U-CAN lymphoma biobank in Sweden: A comparison with nationwide registers. Br J Haematol. 2024;205(5):1794–803.

3.	Abrey LE, Ben-Porat L, Panageas KS, Yahalom J, Berkey B, Curran W, et al. Primary central nervous system lymphoma: the Memorial Sloan-Kettering Cancer Center prognostic model. J Clin Oncol. 2006;24(36):5711–5.

4.	Ferreri AJM, Illerhaus G, Doorduijn JK, Auer DP, Bromberg JEC, Calimeri T, et al. Primary central nervous system lymphomas: EHA-ESMO Clinical Practice Guideline for diagnosis, treatment and follow-up. Hemasphere. 2024;8(6):e89.

5.	Ferreri AJ, Reni M, Foppoli M, Martelli M, Pangalis GA, Frezzato M, et al. High-dose cytarabine plus high-dose methotrexate versus high-dose methotrexate alone in patients with primary CNS lymphoma: a randomised phase 2 trial. Lancet. 2009;374(9700):1512–20.

6.	Abrey LE, Batchelor TT, Ferreri AJ, Gospodarowicz M, Pulczynski EJ, Zucca E, et al. Report of an international workshop to standardize baseline evaluation and response criteria for primary CNS lymphoma. J Clin Oncol. 2005;23(22):5034–43.
