# Supplementary material for: No association between temozolomide maintenance and survival after first‐line treatment in primary CNS lymphoma
Source: Hemasphere. 2026 Aug 17;10(8):e70445. doi: 10.1002/hem3.70445 (PMC13479465; doi:10.1002/hem3.70445)
Supplement: Supplementary file 2 [file HEM3-10-e70445-s002.docx]

**Supplementary Table 1.** Patient characteristics for Uppsala and Lund subgroups.

|  | | **All** | **Uppsala** | **Lund** | p-value |
| --- | --- | --- | --- | --- | --- |
| **Number of patients** | | 110 | 60 | 50 |  |
| **Sex**  **Male (%); Female (%)** | | 58 (53)**;**  52 (47) | 32 (53)**;**  28 (47) | 26 (52)**;** 24 (48) | 1.0 |
| **Median age**  **(range, standard deviation)** |  | 67  (29-82, 10.6) | 67  (31-82, 9.8) | 65  (29-76, 11.2) | 0.02 |
| **Age groups per years,**  **N (%)** | **29-49**  **50-59**  **60-69**  **≥70** | 10 (9)  13 (12)  45 (41)  42 (38) | 4 (7)  5 (8)  26 (43)  25 (42) | 6 (12)  8 (16)  19 (38)  17 (34) | 0.42 |
| **Median year at diagnosis**  **(range)** | | 2014  (2009-2022) | 2015  (2010-2022) | 2014  (2009-2018) | 1.0 |
| **Site of disease**  **N (% ^(a)^)** | **Deep structures** | 77 (73) | 32 (57) | 45 (90) | 1.6×10⁻⁴ |
|  | **Unknown** | 4 | 4 | 0 |  |
| **ECOG performance status (WHO) (% ^(a)^)** | **0** | 24 (30) | 12 (21) | 12 (54) |  |
|  | **1** | 36 (46) | 27 (47) | 9 (41) |  |
|  | **2** | 7 (9) | 6 (11) | 1 (5) |  |
|  | **3** | 8 (10) | 8 (14) | 0 (0) |  |
|  | **4** | 4 (5) | 4 (7) | 0 (0) | 0.03 |
|  | **Unknown** | 31 | 3 | 28 |  |
| **MSKCC score  N (% ^(a)^)** | **1** | 5 (6) | 2 (3) | 3 (14) |  |
|  | **2** | 56 (71) | 38 (67) | 18 (81) |  |
|  | **3** | 18 (23) | 17 (30) | 1 (5) | 0.02 |
|  | **Unknown** | 31 | 3 | 28 |  |
| **HD-MTX regimen**  **N (% ^(a)^) ^(b)^** | **Total** | 84 (76) | 34 (57) | 50 (100) |  |
|  | **MATRix** | 11 (10) | 6 (10) | 5 (10) |  |
|  | **MPV** | 62 (56) | 21 (35) | 41 (82) |  |
|  | **MATRix + MPV** | 5 (5) | 2 (3) | 3 (6) |  |
|  | **Other** | 6 (5) | 5 (8) | 1 (2) | 0.0791 |

| **First-line**  **induction response  N (% ^(a)^)** | **CR** | 51 (53) | 24 (48) | 27 (59) |  |
| --- | --- | --- | --- | --- | --- |
|  | **PR** | 23 (24) | 9 (18) | 14 (30) |  |
|  | **SD** | 6 (6) | 2 (4) | 4 (9) |  |
|  | **PD** | 7 (7) | 6 (12) | 1 (2) |  |
|  | **No treatment** | 9 (9) | 9 (18) | 0 (0) | 0.2 |
|  | **Unknown** | 14 | 10 | 4 |  |
| **Completed 3 cycles of HD-MTX** | | 66 (60) | 26 (43) | 40 (80) | 9.6×10⁻⁵ |
| **Rituximab N (%) ^(b)^** | | 60 (55) | 40 (67) | 20 (40) | 0.007 |
| **WBRT N (%) ^(b)^** | | 38 (35) | 22 (36) | 16 (32) | 0.7 |
| **ASCT N (%) ^(b)^** | | 14 (13) | 9 (15) | 5 (10) | 0.6 |
| **TMZ induction N (%) ^(b)^** | | 21 (19) | 21 (35) | 0 (0) | 5.8×10⁻⁷ |
| **TMZ maintenance** | | 34 (31) | 20 (33) | 14 (28) | 0.68 |
| **Median number of temozolomide maintenance cycles (range)** | | 10  (1-13) | 10  (1-13) | 8.5  (1-13) | 1.0 |
| **Best supportive care** | | 9 (8) | 9 (15) | 0 (0) | 0.004 |
| **Dead** | | 78 (71) | 45 (75) | 33 (66) | 0.40 |
| **Median follow-up time in months**  **(range)** | | 29.09  (0.33-200.97) | 12.67  (0.33-163.45) | 59.47  (2.27-200.97) | 0.0003 |

The number of patients with a positive (“Yes”) value for each binary variable is presented as numbers with percentages in parentheses. Patients without the variable were considered negative (“No”). For variables with missing data, the number of patients with unknown status is also presented.

Abbreviations: ECOG = Eastern Cooperative Oncology Group; WHO = World Health Organization; HD-MTX = High-dose methotrexate; ASCT = Autologous stem cell transplantation; MSKCC = Memorial Sloan Kettering Cancer Center score; MATRix = methotrexate, cytarabine, thiotepa, rituximab; MPV = methotrexate, procarbazine and vincristine; CR = complete response; PR = partial response; SD = stable disease; PD = progressive disease.

^(a)^ Percentage from patients with known status

^(b)^ Received as part of the first-line treatment

**Supplementary Figure 1.** Overall survival stratified according to patient characteristics.

(A) Entire cohort; (B) Age groups; (C) ECOG performance status; (D) Sex.

Abbreviations: ECOG = Eastern Cooperative Oncology Group

**Supplementary Figure 2.** Overall survival stratified for treatments.

Overall survival stratified according to administration of high-dose methotrexate (HD-MTX) in all patients (A), and according to completion of HD-MTX induction (defined as receiving three or more treatment cycles) among those treated with HD-MTX (B). Overall survival for all patients stratified according to receipt of autologous stem cell transplantation (ASCT) (C), first-line temozolomide induction compared with best supportive care (BSC) (D), and first-line whole-brain radiotherapy (WBRT) (E). Overall survival stratified according to radiological response to induction and/or consolidation treatment in all patients (F).

Abbreviations: HD-MTX = high-dose methotrexate; WBRT = whole-brain radiotherapy; ASCT = autologous stem cell transplantation; BSC = best supportive care; CR = complete response; PR = partial response; SD = stable disease; PD = progressive disease.

**Supplementary Figure 3. Survival based on Temozolomide maintenance in relation to WBRT and radiological response.**

Overall survival stratified for temozolomide maintenance treatment without WBRT (A) or with WBRT (B). Overall survival stratified for radiological response (C) after first line induction and temozolomide maintenance for all the patients who completed three cycles of high-dose Methotrexate (HD-MTX) treatment.

Abbreviations: WBRT = whole brain radiotherapy HD-MTX = high-dose methotrexate, TMZ = Temozolomide, CR = complete response.
